# Dramatic inhibition of osteoclast sealing ring formation and bone resorption in vitro by a WASP-peptide containing pTyr294 amino acid

**DOI:** 10.1186/1750-2187-3-4

**Published:** 2008-02-20

**Authors:** Tao Ma, Venkatesababa Samanna, Meenakshi A Chellaiah

**Affiliations:** 1 Department of Biomedical Sciences, Dental School, University of Maryland, Baltimore, MD 21201, USA; 2 Gazes Cardiac Research Institute at Medical University of South Carolina, Charleston, SC 29403, USA

## Abstract

Wiskott Aldrich Syndrome protein (WASP) has a unique regulatory role in sealing ring formation and bone resorption in osteoclasts. Here, using the TAT-transduction method, we show the possible role of WASP domain(s) in sealing ring formation and bone resorption. Transduction of TAT-fused full-length WASP peptide induced Arp2/3 complex formation, F-actin content, sealing ring formation and bone resorption. Transduction of WASP peptides containing basic, verpolin-central, pTyr294, and proline-rich regions inhibited the processes listed above at various levels. The ability to resorb bone by WASP peptides containing basic, verpolin-central, and proline-rich regions was reduced and the resorbed area matched the size of the sealing ring. However, osteoclasts transduced with WASP peptide containing pTyr294aa demonstrated the following: a) a considerable decrease in the interaction and phosphorylation of c-Src with endogenous WASP; b) total loss of sealing ring-like structures; c) formation of actin-rich patches at the peripheral edge that contains filopodia-like projections; d) reduced capacity for bone resorption in vitro. These findings suggest that modulation of phosphorylation state of pTyr294aa assists in integrating multiple signaling molecule and pathways that partake in the assembly of sealing ring.

## Background

Osteoclasts are highly motile multinucleated giant cells actively involved in bone resorption. Osteoclast function depends on dynamic regulation of the actin cytoskeleton to accomplish its ordered cycles of movement and attachment during bone resorption [1-6]. Osteoclasts attach themselves to bone matrix surfaces through unique cell adhesion structures known as podosomes [4-6]. Changes in podosome assembly/disassembly allow for osteoclast migration, adhesion, and bone resorption. Sealing ring formation is considered a marker of osteoclast activation for bone resorption. Changes in cell shape, organization of podosomes for cell migration, and formation of actin ring (also known as sealing ring) for bone resorption require regulated assembly/disassembly of actin filaments.

Structural dynamics of actin cytoskeleton are dependent on the function of actin-binding proteins, which comprise many proteins with distinct properties, such as severing, capping, cross-linking, and nucleation. We have previously shown that gelsolin, an actin capping/severing protein, plays a key role in podosome assembly/disassembly and osteoclast migration. Gelsolin deficient (Gsn-/-) osteoclasts failed to display distinct podosomes. Mechanisms of cell attachment substituting for podosomes were expressed in Gsn-/osteoclasts. However, these adhesion structures failed to support osteoclast motility [4].

Although Gsn-/- osteoclasts failed to exhibit distinct podosome structures, they contained the sealing ring. The presence of WASP in the sealing ring of Gsn-/- osteoclasts suggests the likelihood of the role of WASP in the bone resorption of osteoclasts [7]. Therefore, Gsn-/- osteoclasts are capable of resorbing bone, but the resorbed areas are small owing to the absence of podosomes and the resulting hypomotile nature of osteoclast [4]. It was found that osteoclasts from WASP-null mice were markedly depleted of podosomes and failed to exhibit sealing ring. Complementation of WASP with WASP-fusion proteins restores normal cytoskeletal architecture [8].

WASP stimulates actin filament nucleation by Arp2/3 complex [9]. WASP consists of multiple domains that can bind to a variety of signaling proteins. Basic region (BR) and GTPase binding domain (GBD) of WASP bind to phosphatidylinositol 4, 5 bisphosphate (PtdIns P2) and Cdc42, respectively [10]. WASP binds in vitro to SH3 domains from c-Src family kinases. A domain rich in polyproline sequence (Pro) binds to a variety of signaling molecules, including Hck, Nck, FAK, Src, and Tec-family kinases [11-14]. Phosphorylation of WASP increases its stability and affinity for the Arp2/3 complex via the C-terminal Verpolin-like, Central, and Acidic domain (VCA domain) [15]. Arp2/3 complex nucleates branched actin filaments in the process of actin polymerization [16,17]. Microinjection of VCA and VC domains resulted in a significant increase of cellular polymerized actin in macrophages [18,19]. These constructs can compete with endogenous WASP and disrupt the binding of Arp2/3 complex. This resulted in preventing the formation of Arp2/3-dependent actin-enriched podosome structures in macrophages [19].

WASP was shown to be imperative in the assembly of sealing ring in osteoclasts. WASP integrates signals from Rho, Cdc42, and kinase(s) to bind to the Arp2/3 complex and WASP-Arp2/3 mediated actin polymerization [2,20]. This sequence of events appears to be important in the formation of sealing ring. Rho-mediated PtdIns P2 interaction with WASP has a role in the activation and membrane targeting of WASP. Subsequent interaction of Cdc42 and Arp2/3 complex with WASP enhances cortical actin polymerization in the process of sealing ring formation during bone resorption [2]. WASP is also tyrosine phosphorylated following transfection with constitutively active c-Src or treatment with osteopontin (OPN) [20]. Both tyrosine kinase(s) and phosphatase PTP-PEST coordinate the formation of sealing ring and bone resorption function of osteoclasts [20]. Osteoclasts transfected with SiRNA to WASP attenuates osteoclast bone resorption owing to failure in the organization of sealing rings [2].

Structure-function analysis of WASP facilitated defining the role of distinct domains in actin polymerization [11,15,21,22]. Transduction of TAT-fused gelsolin fragments, containing phosphoinositide binding domains (PBDs) or full-length gelsolin resulted in the disruption of actin remodeling processes, which are essential for podosome assembly and sealing ring formation. Transduction of TAT-fused gelsolin fragments containing PBDs exhibited a dominant negative effect in the formation of WASP-Arp2/3 complex, indicating the role of phosphoinositides in WASP activation [23]. We have used similar strategy to determine the WASP domain (motif) that has a key role in osteoclast function; we cloned different WASP motifs in HA-TAT expression vector. The purified TAT-fused WASP fragments containing various domains were transduced into osteoclasts. The effects of these domains on Arp2/3 complex formation, c-Src interaction with endogenous WASP, F-actin content, sealing ring formation and bone resorption were determined. A significant decrease in F-actin content, sealing ring formation, and bone resorption was observed in osteoclasts transduced with WASP peptides containing pTyr294aa (pTyr294 in mouse and pTyr291 in human WASP), BR, VCA, and pro-rich regions. However, the inhibition was more prominent with a WASP peptide containing pTyr294aa. Our observations suggest that phosphorylation of pTyr294 by kinase(s) regulates sealing ring formation by WASP-Arp2/3 complex-mediated actin polymerization.

## Methods

### Reagents

Antibodies to WASP, HA, Arp2, c-Src and c-Src pTyr418 were purchased from Santa Cruz Biotechnology, Inc. (Santa Cruz, CA). Rhodamine phalloidin was bought from Sigma-Aldrich chemicals (St.Louis, MO). Ni-NTA sepharose beads were purchased from Pharmacia chemicals. Acrylamide-bisacrylamide solution and protein estimation reagent were obtained from Bio-Rad.

### Cell culture

C57/BL6 mice were used for in vitro osteoclast generation. Bone marrow cells isolated from five mice were cultured into 100 mm dishes with 20 ml α-MEM medium, supplemented with 10% fetal bovine serum (α-10). After culturing for 24 h, non-adhered cells were layered on histopaque-1077 (Sigma) and processed as described previously [20]. Cells were cultured with the appropriate concentrations of mCSF-1 (10 ng/ml; R and D chemicals, IN) and RANK-L (55–75 ng/ml). After three days in culture, media were replaced with fresh cytokines. The multinucleated osteoclasts were seen from day four onwards.

### Cloning of WASP fragments

Bacterial expression constructs coding various HIV-TAT fusion peptides of WASP were generated by PCR method. cDNA from mouse osteoclast and full length WASP in pGEX-2T were used as PCR templates. WASP constructs were generated from mouse cDNA library by using the following primers: *Full length (FL):*5'catgccatgggcatgagtgggggcccaatgggaggaag3' and 5'acatgcatgcac ttatcagtcatcccattcatcatcttcatc3'; *Basic region (BR):*5'catgccatgggcgctgataagaaacg ctcag3' and 5'acatgcatgcactt aatcagctttgctgatcttc3'; *GBD and Pro Rich domain (GP):*5' catgccatgggctcagggaaga agaagatcag3' and 5'acatgcatgcacttaattcccggagctgggcggcggtg3'; p*Tyr294 containing fragment (pTyr):*5'catgccat gggcggctgggatccccagaatg3' and 5'acatgcatgcacttactcttggc gcctcatctcctg 3'; *Proline Rich domain (PRO); *5'catgccatgggccaggagatgaggcgccaagag3' and 5'acatgcatgcacttacactggagtaggagggagcgggggag3'; *Verpolin, central domain (VC):*5'catgccatgggc ctggcccctggtgggggtc3' and 5'acatgcatgca cttactccccttcgtcggaggagtg3'; *Verpolin-like, central, and acidic domain (VCA):*5'catgccatgggcgtgcctgccgggggcctg3' and 5'acatgcatgcacttatcagtcatcccattc atcatcttcatc3'. The PCR product was digested with Nco I and Sph I restriction enzymes and inserted into the HA-TAT fusion vector, which was cut with the same restriction enzymes. The sequences of all the clones were confirmed for reading frame by DNA sequencing.

### Purification of TAT-fused WASP proteins

Escherichia coli (BL-21 strain from Invitrogen, Carlsbed, CA) was used to transform HA-TAT vector containing the above-mentioned WASP constructs. Protein was purified using Ni-NTA column as described previously [24]. Purified proteins were subjected to SDS-PAGE followed by Western analysis with a hemagglutinin (HA) antibody and Coomassie blue staining to determine the molecular weight and homogeneity [23]. HA-TAT vector (8–10 kDa) and Herplex simplex virus thymidine kinase (Hsv-TK; 42 kDa) proteins were used as controls for transduction experiments [24].

### Preparation of osteoclast lysate after various treatments, immunoprecipitation, and immunoblotting

Following transduction with various proteins, osteoclasts were washed three times with cold PBS and lysed in RIPA buffer (10 mM Tris-HCl, pH 7.2, 150 mM NaCl, 1% deoxycholate, 1% Triton ×-100, 0.1% SDS, 1% aprotinin and 2 mM PMSF) and centrifuged at 15,000 rpm for 15 min at 4°C. Protein contents were measured using the Bio-Rad protein assay reagent. Equal amounts of lysate proteins were used for immunoprecipitation with antibody to WASP or HA. Immunoprecipitation and immunoblotting were performed as described previously [25].

### Actin staining

Osteoclast precursors (10^5 ^cells) were seeded on dentine slices and cultured for 36–48 h. Prior to staining; osteoclasts were washed three times with PBS containing 5 mM EGTA (PBS/EGTA). Osteoclasts were fixed with 3% paraformaldehyde in PBS/EGTA for 20 min. Subsequently, cells were permeablized with 0.1% Triton ×-100 in PBS/EGTA for 5 min. Osteoclasts were incubated with rhodamine phalloidin (Sigma; 1:1000 dilution) for 30 min. at 37°C or overnight at 4°C as described earlier [1]. The cells on dentine slices were washed and mounted on a slide in a mounting solution (Vector laboratories, Inc., Burlingame, CA) and sealed with nail polish. Immunostained osteoclasts were photographed with a Bio-Rad confocal laser-scanning microscope. Images were stored in TIF image format and processed by the Adobe Photoshop software program (Adobe System Inc., Mountain view, CA).

### Measurement of F-actin content using rhodamine phalloidin binding

On day four, osteoclasts were transduced with 100 nM TAT-fused WASP peptides and incubated for 36–48 h. After transduction, osteoclasts were rinsed three times. Subsequently, cells were fixed, permeablized, and incubated with rhodamine phalloidin (1:200) as described earlier [20]. Cells were extracted with absolute methanol and the fluorescence of each sample was measured with fluorimetry (Bio-RAD spectrofluorometer). Osteoclasts untreated with rhodamine phalloidin were used to determine the background fluorescence of the cells. Ten-fold excess unlabelled phalloidin was used to determine the non-specific binding. The non-specific binding and background fluorescence were subtracted from the total binding to determine the specific binding [26,27].

### Bone resorption assay

Osteoclasts transduced with the indicated TAT-WASP proteins were added to 48-well-containing dentine slices (2 × 10^4 ^cells). After 2 h of adherence, α-MEM medium containing 10% FBS and RANK-L was added. The same medium was replaced with the respective TAT-fused WASP proteins after 24 h. After 48 h, cells were scraped from dentine, and the slices were washed twice with water. Dentine slices were stained with acid hematoxylin (Sigma) and washed with water to remove excess stain. Pits were scanned under confocal microscopy. Images were stored in TIF image format and processed by the Adobe Photoshop software program (Adobe Systems, Inc., Mountain View, CA).

### Data analysis

All values presented are expressed as the mean ± standard error of the mean (SEM) of three or more experiments done at different times normalized to intra-experimental control values. Asterisks were used to graphically indicate the statistical significance. A value of p < 0.05 was considered significant. For statistical comparisons, analysis of variance (ANOVA) was used with the Bonferroni corrections (Instat for IBM, version 2.0; Graphpad software).

## Results

### Expression and purification of WASP proteins and determination of the levels of transduced proteins in osteoclasts

**Figure 1:**
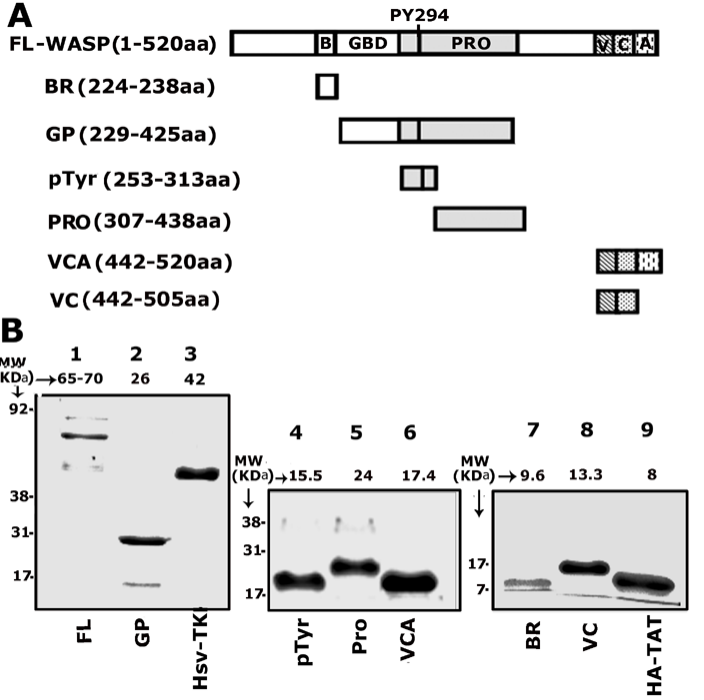
**A. Schematic diagram demonstrating various WASP constructs generated in HA-TAT expression vector**. The domain organization of WASP is shown in full length WASP (WASP-FL). These domains (BR, basic region; GBD, GTPase binding domain; GP, GTPase binding domain and proline rich domain; Pro, proline-rich region; Verpolin-like, central, and acidic domain [VCA]; Verpolin-like, and central domain [VC]) are cloned separately into the HA-TAT expression vector. The number within the parentheses indicates the first and last amino acid of the corresponding WASP peptide. **B**. **SDS-PAGE analysis demonstrates the purified TAT-fused WASP and control (Hsv-TK and HA-TAT) proteins**. TAT-fused proteins were subjected to 8% (lanes 1–3) and 15% (lanes 4–9) SDS-PAGE and stained with Coomassie blue. The numbers on the left of each panel represent the standard molecular weight (MW) markers (kDa). The numbers on the top of each lane indicate the apparent molecular mass (kDa) of the purified protein.

A schematic diagram of WASP constructs cloned into HA-TAT expression vector is shown in Fig. 1A. Cloning was performed as described in the Methods section. Amino acid sequence of each WASP construct is shown within the parentheses. Purified proteins were analyzed by SDS-PAGE and stained with Coomassie blue (Fig. 1B). In an 8% SDS-PAGE gel (Fig. 1B) purified TAT-fused full length (FL)-WASP (65–70 kDa; lane 1), **G**TPase binding and **p**roline rich-domain (GP domain; 26 kDa, lane 2) and human simian virus thymidine kinase protein (Hsv-TK; 42 kDa; lane 3) are shown. The purified TAT-fused WASP peptide containing phospho-tyrosine 294 aa (pTyr, 15.5 kDa, lane 4), proline-rich sequences (Pro; 24 kDa; lane 5) VCA (17.4 kDa; lane 6), basic (BR; 9.6 kDa; lane 7), and VC (13.3 kDa; lane 8) domains are shown in a 15% SDS-PAGE. HSV-TK and vector (6–8 kDa; HA-TAT; lane 9) proteins were used as controls for transduction experiments. Phosphotyrosine 294 aa (pTyr294aa) in mouse WASP (includes the first methionine aa) corresponds to pTyr 291 in human WASP. There are six proline-rich clusters in the TAT-fused WASP-Pro construct (307–428aa). The number given below within the parentheses indicates the proline-rich aa sequences in each cluster: 311–318 (8), 350–355(6), 358–361 (4), 366–372 (7), 379–385 (7), and 390–403 (14).

**Figure 2:**
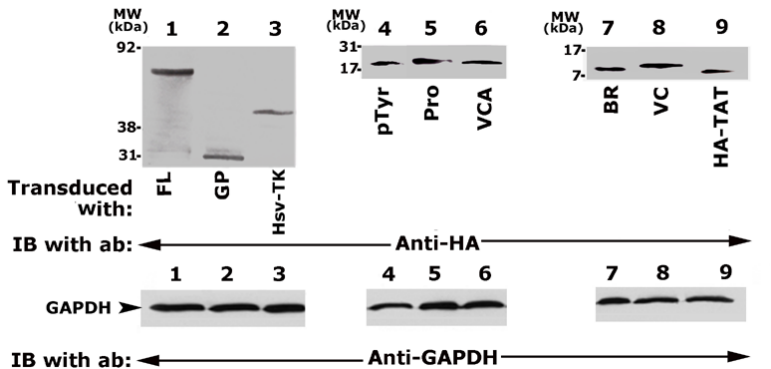
**Immunoblotting analysis of the levels of transduced proteins in osteoclasts with HA-antibody**. Osteoclasts were transduced with the WASP peptides as shown below the figures. Osteoclast lysates (~200 μg) were immunoblotted with an antibody to HA to determine the levels of transduced proteins (Top panel). The immunoblot shown in the top panel was stripped and blotted with a GAPDH antibody for normalization (bottom panel). The results shown are representative of three independent experiments.

Based on the dose-dependent uptake experiment (data not shown), a concentration of 100 nM TAT-fused WASP protein was decided for transduction experiments given below. The uptake of TAT-fused WASP proteins were determined by immunoblotting with an antibody to HA (Fig. 2, top panel). About 200 μg of osteoclast lysate was used for immunoblotting analysis. Western blot analysis with anti-HA demonstrates the levels of transduced peptides in each lane (Fig. 2, top panel). Loading was normalized to the cellular levels of GAPDH (Fig. 2, bottom panel).

### Analysis of the effects of transduction of TAT-fused WASP proteins on the F-actin content

**Figure 3:**
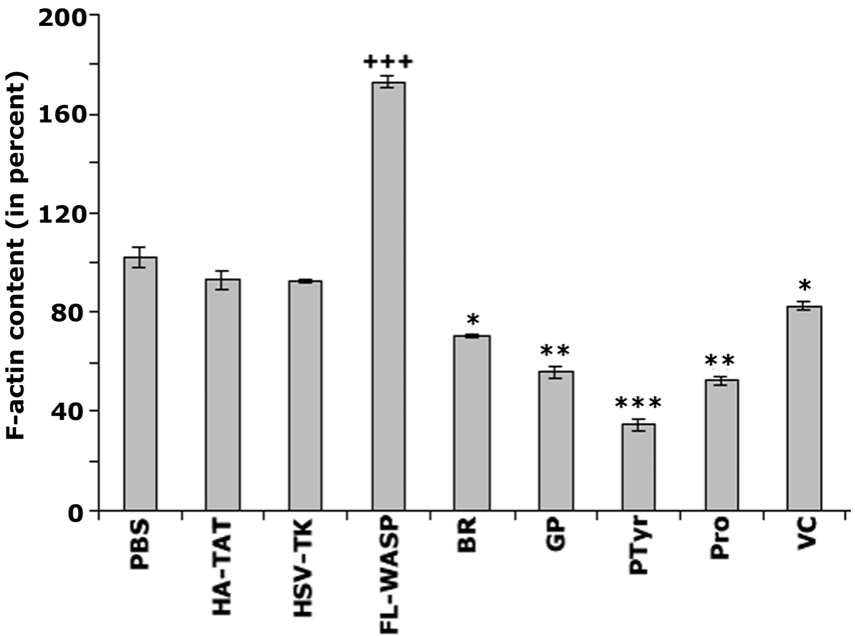
**Measurement of F-actin content by rhodamine phalloidin binding in osteoclasts**. Cells were grown in 24 well-tissue culture plates, and three to four wells were used for each treatment indicated in the figure. The results presented are mean ± SE for three experiments. *** p < 0.0001 versus PBS-treated as well as HA-TAT, Hsv-TK, and FL-WASP transduced osteoclasts; ^+++ ^p < 0.001, ** p < 0.01, *p < 0.05 versus PBS-treated as well as HA-TAT and Hsv-TK transduced osteoclasts.

VCA domain of WASP binds actin monomers by the V domain, and the Arp2/3 complex through the C and A domains. This interaction increases actin polymerization in vivo and in vitro [2,15,19,28]. We examined the effects of transduction of various TAT-fused WASP peptides on the F-actin content in osteoclasts (Fig. 3). A significant increase in the F-actin content was observed in osteoclasts transduced with FL-WASP. Osteoclasts treated with PBS- or transduced with HA-TAT and Hsv-TK exhibited the basal level F-actin content. A decrease in the F-actin content was observed in osteoclasts transduced with basic (BR), GP, pTyr294aa, Pro, and VC domains. The decrease in the F-actin content was more pronounced in osteoclasts transduced with TAT-fused WASP fragment containing pTyr294aa.

### Analysis of the effects of transduction of TAT-fused WASP proteins on the interaction of Arp2 with WASP

**Figure 4:**
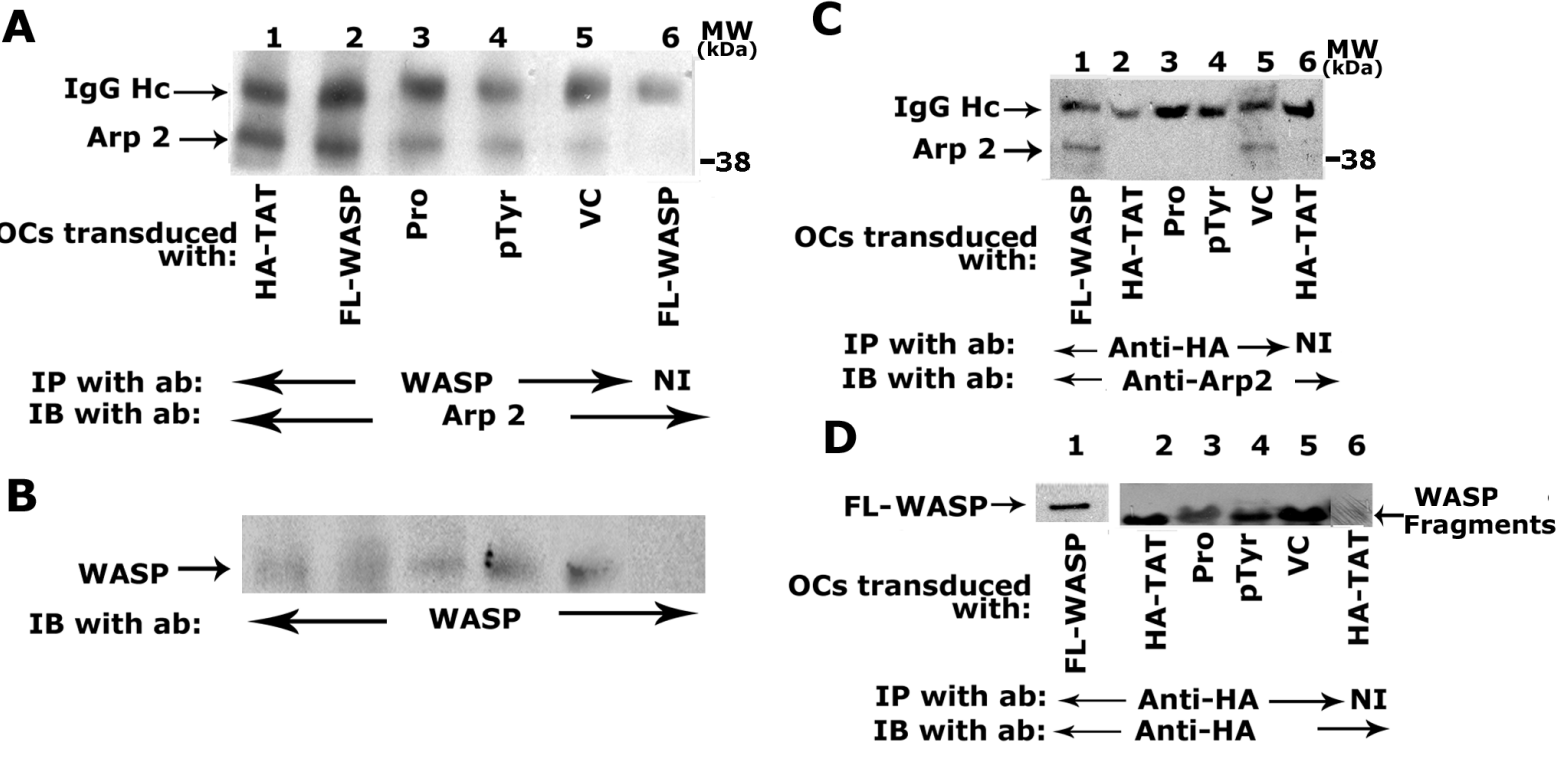
**Analysis of the interaction of Arp2 with the endogenous WASP and transduced peptides**. Osteoclasts were transduced with the WASP peptides as shown below the figures. Lysates were immunoprecipitated with a WASP (A and B) or HA (C and D) antibody. A and B: WASP immunoprecipitates were first immunoblotted with an antibody to Arp2 (A) and subsequently stripped and blotted with an antibody to WASP (B). C and D: HA-immunoprecipitates were divided into two halves; one half was immunoblotted with an Arp 2 antibody (C) and the other half was subjected to 8% (D, lane 1) and 15% (D, lanes 2–5) SDS-PAGE. Immunoblotting was performed with a HA-antibody (D) to detect the levels of transduced proteins immunoprecipitated in each lane. Immunoprecipitation with a nonimmune serum is shown in lane 6 (A-D). The results represent one of three experiments performed from three separate osteoclast preparations.

Subsequently, we proceeded to analyze the effects of various TAT-fusion proteins on Arp2 interaction with endogenous WASP (Fig. 4A) and transduced TAT proteins (Fig. 4C). Osteoclast lysates were immunoprecipitated with WASP antibody and immunoblotted with an antibody to Arp2 (Fig. 4A). Interaction of Arp2 with the immunoprecipitated endogenous WASP protein was observed in osteoclasts transduced with HA-TAT vector protein and FL-WASP (Fig. 4A, lanes 1 and 2). This interaction is considerably reduced with endogenous WASP in osteoclasts transduced with TAT-fused WASP peptides containing the following domain: proline-rich (Fig. 4A, lane 3), pTyr294aa (lane 4), and VC (lane 5). However, the decrease was more in osteoclasts transduced with WASP peptides containing pTyr294aa (lane 4) and VC (lane 5). Stripping and reprobing of this blot with an antibody to WASP demonstrated the WASP level in each immunoprecipitates (Fig. 4B).

Since VC transduced osteoclasts exhibited reduced levels of Arp2 interaction with endogenous WASP, osteoclast lysates were immunoprecipitated with a HA-antibody to determine the interaction of Arp2 with the transduced peptides. HA immunoprecipitates were divided into two halves. The first half was subjected to 8% SDS-PAGE, immunoblotted with Arp 2 antibody (Fig. 4C). Binding of Arp 2 with transduced FL-WASP (Fig. 4C, lane 1) and VC domain (4C, lane 5) was observed. The observed decrease in the F-actin content in osteoclasts transduced with VC domain (Fig. 3) may be caused by the competitive binding of Arp2 with these fragments (Fig. 4C, lanes 1 and 5). The significant increase in F-actin content in FL-WASP transduced osteoclasts (Fig. 3) may be due to the combined effects of endogenous and transduced WASP in the actin polymerization process. Transduction of WASP peptides containing pTyr294aa or Pro-rich sequences also blocked Arp-2 binding despite the fact that these peptides do not have any binding site for Arp2/3 complex. We have recently shown that the phosphorylation of WASP protein, as well as its interaction with signaling proteins, is critical for actin polymerization [20]. One possible reason could be that the phosphorylation and interaction of signaling protein with endogenous WASP may be competitively blocked by these peptides. As a result of this interaction, the stability of active WASP, its interaction with Arp2 and actin polymerization (Figs. 3 and 4) may have decreased. The second half of the HA-immunoprecipitates was subjected to 8% (Fig. 4D, lane 1) and 12% (lanes 2–7) SDS-PAGE. Immunoblotting with a HA-antibody demonstrated the presence of transduced TAT-proteins in the osteoclast lysate. Immunoprecipitation with a non-immune serum is shown in lane 6 (Fig. 4A–D).

### Analysis of the effects of transduction of various TAT-fused WASP peptides on the interaction of c-Src with endogenous WASP

**Figure 5:**
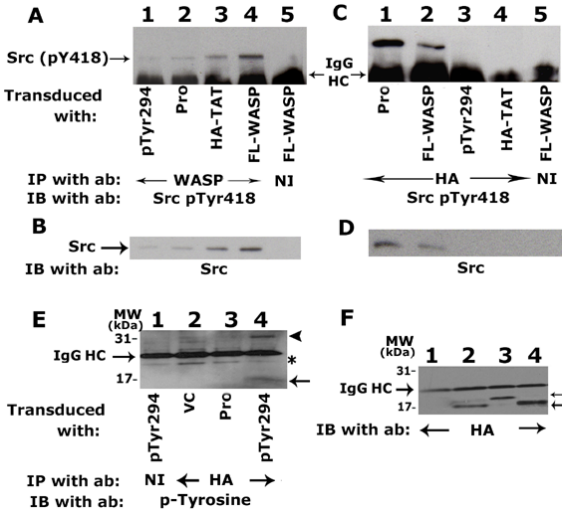
**Analysis of the interaction of c-Src with the endogenous WASP and transduced peptides**. A-D: Osteoclasts were transduced with the WASP peptides as shown below the figures. Lysates were immunoprecipitated with a WASP (A and B) or HA (C and D) antibody. Immunoprecipitates were first immunoblotted with an antibody to Src pTy418 (A and C). Subsequently, blots were stripped and blotted with an antibody to c-Src (B and D) to detect the levels of c-Src coprecipitated with WASP or HA immunoprecipitates. Immunoprecipitation with a non-immune serum (NI) is shown in lane 5 (A-D). E and F: HA-immunoprecipitates were subjected to 15% SDS-PAGE. Immunoblotting was performed with a phosphotyrosine (p-Tyrosine; panel E) to detect the phosphorylation levels of transduced proteins. Subsequently, blot was stripped and blotted with a HA- antibody (F) to determine the levels of transduced proteins immunoprecipitated in each lane. An asterisk in Fig. 5E indicates coprecipitation of a non-specific protein with HA and non-immune (NI) immunoprecipitates (lanes 1–4). Immunoprecipitation with a non-immune serum (NI) is shown in lane 1 (E and F). The results represent one of three experiments performed from three separate osteoclast preparations.

We have previously demonstrated that tyrosine phosphorylation of WASP and associated proteins are in part dependent on the c-Src kinase activity [20]. We hypothesized that transduction of WASP fragments containing proline rich-sequences and pTyr294 sequence would competitively reduce the interaction of c-Src with endogenous WASP. The ability of the transduced peptides containing proline rich and pTyr294aa sequences to interfere the binding of c-Src with endogenous WASP was investigated as shown in Figure 5. Immunoprecipitates made with an antibody to WASP (Figure 5A) or HA (Figure 5C) were immunoblotted with a Src-pTyr418 antibody. Subsequently, these blots were stripped and reprobed with a c-Src antibody (Figures 5B and 5D).

Coprecipitation of c-Src was observed with WASP immunoprecipitates in osteoclasts transduced with vector (HA-TAT) and various WASP peptides (Fig. 5A and 5B). The phosphorylation (Fig. 5A) and protein (Fig 5B) levels of c-Src are more in osteoclasts transduced with FL-WASP (lane 4) than the basal level observed in osteoclasts transduced with HA-TAT (lane 3). The increase in Src level in FL-WASP transduced osteoclasts may be due to that WASP antibody precipitates both the endogenous and transduced WASP protein. Phosphorylation (A, lanes 1 and 2) and protein (B, lanes 1 and 2) levels of c-Src associated with endogenous WASP are very minimal in osteoclasts transduced with WASP fragments containing pTy294 aa and proline-rich sequences.

The reduced interaction of c-Src with endogenous WASP (Figure 5A and 5B; lane 2) in OCs transduced with TAT-fused proline sequences was parallel with an increase in the association of c-Src with the transduced peptide in the immunoprecipitation performed with a HA-antibody (Fig. 5C; lane 1). An increase in the phosphorylation (Fig. 5C, lane 1) and protein (Fig 5D, lane 2) levels of c-Src may be due to the presence of six proline-rich clusters in this fragment. Interaction of c-Src with proline-rich sequence (Figure 5D, lane 1) is more than the level observed with FL-WASP peptide (Fig. 5D, lane 2). WASP fragment containing pTyr294 has only two proline aa sequences of the first cluster. Therefore, interaction of c-Src with this fragment was very negligible or not observed (Fig. 5C and 5D, lane 3). The interaction c-Src with the transduced proline sequences and FL-WASP is specific as immunoprecipitation with a HA- antibody in osteoclasts transduced with vector protein HA-TAT failed to exhibit coprecipitation of c-Src. These observations confirm the previously demonstrated specific interaction of c-Src with WASP [20].

Inhibition of Src interaction with endogenous WASP in cells transduced with pTyr294 fragment suggests that this peptide may have the ability to block this interaction through competitive inhibition of the phosphorylation of endogenous WASP. Subsequently we addressed the question whether the transduced WASP fragment containing pTyr294 is phosphorylated in vivo (Fig. 5E). Lysates made from osteoclasts transduced with WASP peptides containing VC domain (lane 2), proline-rich region (lane 3), pTyr294aa (lane 4) were immunoprecipitated with a HA antibody and subjected to 15% SDS-PAGE. Immunoblotting analysis with a p-Tyrosine antibody demonstrated coprecipitation and phosphorylation of proteins in osteoclasts transduced with WASP peptide containing pTyr294. Protein with a molecular mass ~31 kDa (Fig. 5E lane 4, indicated by an arrow head) and the transduced pTyr294 peptide (~15.5 kDa; indicated by an arrow) were observed (Fig. 5E, lane 4). Immunoprecipitation with a non-immune serum is shown in lane 1 (E and F). Subsequently, this blot was stripped and reprobed with a HA antibody in order to characterize the transduced proteins coprecipitated with HA immunoprecipitates (Fig. 5F). WASP peptides containing VC (13.3 kDa, lane 2), proline-rich sequences (24 kDa; lane 3) and p-Tyr294 aa (15.5 kDa, lane 4) were observed. Overall, these results (Fig. 5) suggest that phosphorylation of WASP on pTyr294 is critical for the subsequent interaction of c-Src and other signaling molecules with the proline-rich region of WASP.

### Analysis of the effects of various transduced TAT-fused WASP peptides in the formation of sealing ring and bone resorption in osteoclasts

**Figure 6:**
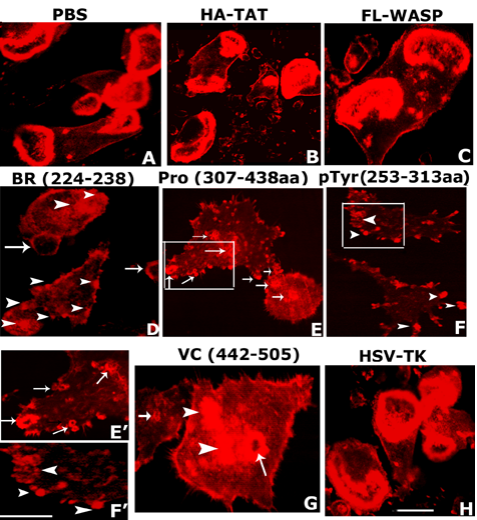
**The effects of transduction of various TAT-fused WASP peptides on sealing ring formation**. Osteoclasts were transduced with indicated TAT-fused peptides or treated with PBS as a control. Confocal microscopy images of osteoclasts stained for actin are shown. Sealing ring was observed in osteoclasts treated with PBS (A) or transduced with HA-TAT (B), FL-WASP (C), and HSV-TK. Sealing ring formation is reduced in osteoclasts transduced with WASP peptides consisting of BR (D), Proline-rich region (E), pTyr amino acid (F), and VC domain (G). The areas that are magnified in E' and F' are shown with a white box in Figs E and F. Arrows and arrowheads indicate small ring-like structures and actin aggregates or patches, respectively (D, E, F, E', F', and G'). Scale Bar: 50 μm. The results shown are representative of three independent osteoclast preparations and experiments.

**Figure 7:**
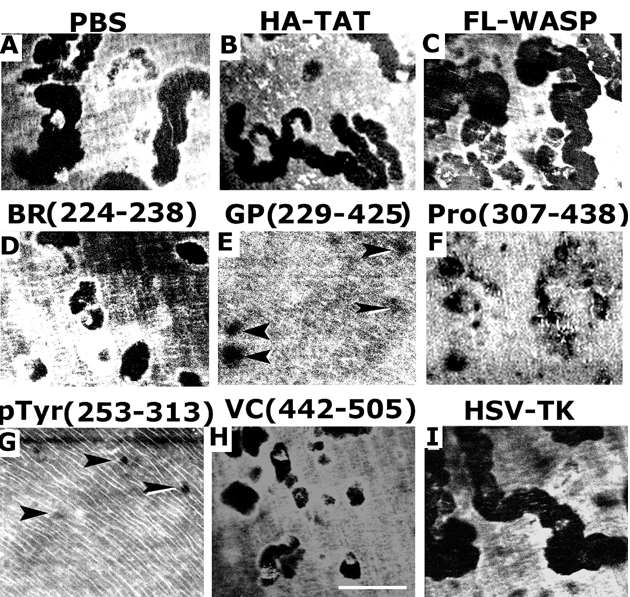
**The effects of TAT-fused WASP peptides transduction on osteoclast bone resorption**. Confocal images of the resorption pits are shown. Osteoclasts were transduced with indicated TAT-fused peptides (B-I) or treated with PBS (A) for 48 h. Pits were scanned under confocal microscopy. Resorption pits were seen as dark spots. These results represent one of three separate experiments performed with the same results. Scale bar-25 μM

We then addressed the question whether increasing the intracellular levels of WASP peptides would reduce sealing ring formation (Fig. 6) and bone resorption (Fig. 7) in osteoclasts. Osteoclasts transduced with the indicated TAT-fused WASP peptides (Fig. 6) were plated on dentine slices and incubated for 36–48 h. Staining of osteoclasts with rhodamine phalloidin for actin, exhibited sealing rings in osteoclasts treated with PBS (Fig. 6A) or transduced with HA-TAT (B), FL-WASP (C), and HSV-TK (H). Sealing ring formation was either reduced or not observed at all in osteoclasts transduced with WASP peptides containing BR (D), proline-rich (E), pTyr294aa (F), and VC (G) sequences. Actin clumps and smaller size sealing rings were observed in the center and at the periphery of the osteoclasts transduced with BR (D), pro (E), and VC (G) domains of WASP. Transduction of proline-rich containing WASP peptide induced tiny nascent actin ring throughout the osteoclasts (E). However, osteoclasts transduced with the WASP peptide containing pTyr294aa (F) exhibited, instead of sealing ring, actin-enriched clusters at the periphery of the osteoclasts. These punctate adhesion-like structures enriched in actin clumps were observed at the edges of filopodia-like projections. These osteoclasts were spread out and adhered well with the dentine slices (F). Areas marked by white square (Fig. E and F) are shown at higher magnification in E' and F'. Arrows and arrowheads indicate the small ring-like structures and actin patches, respectively (D, E, F, G, E', and F'). Reduced actin staining in osteoclasts transduced with peptides containing BR (D), pro (E), pTyr294 (F), and VC (G) agree with the decreased F-actin content shown in Fig. 3. Untransduced (A) as well as transduced osteoclasts with HA-TAT (B) and HSV-TK (H) were used as controls.

**Figure 8:**
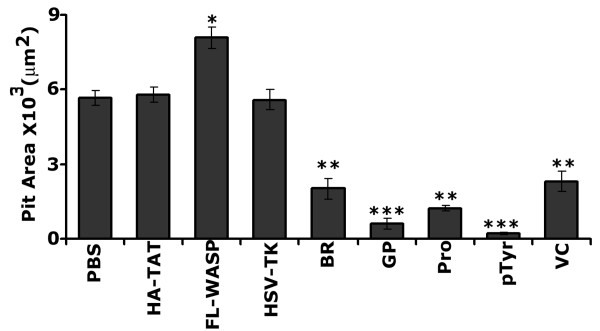
**Quantification of resorption pit generated in vitro by osteoclasts after various treatments**. About 20–25 pits/slice from each experiment were scanned to determine pit area. Group data of pit area from confocal microscopy of pits on multiple dentine slices from three osteoclast preparations are shown. ***p < 0.0001 vs. PBS-treated as well as HA-TAT, Hsv-TK, and FL-WASP transduced osteoclasts; **p < 0.001; * p < 0.05 vs. PBS-treated as well as HA-TAT, Hsv-TK transduced osteoclasts.

We subsequently determined the bone resorption activity of osteoclasts treated with PBS (Fig. 7A) or transduced with the indicated TAT- proteins (Fig. 7, B–I). Quantification of resorption pits (expressed as pit area in μm^2^) generated in vitro by osteoclasts is shown in Fig. 8. Osteoclasts transduced with HA-TAT (B) or HSV-TK (I) and treated with PBS (A) were used as controls. An increase in pit area was observed in osteoclasts transduced with FL-WASP as compared to those transduced and treated with controls (A, B, and I). Inhibition of bone resorption was observed in osteoclasts transduced with TAT- WASP peptides containing BR (D), GP (E), Pro (F), pTyr294aa (G), and VCA (H). GP domain of WASP peptide contains GTPase binding, pTyr294, and pro-rich regions. This fragment was used in this assay to confirm the effects of WASP peptide containing pTyr294 aa. It has significant inhibitory effect on osteoclast bone resorption. However, the decrease was more apparent in osteoclasts transduced with WASP peptide containing pTyr294 aa (G). Bone resorption activity was observed in the following order of the pit area (in μm^2^): FL-WASP > PBS = HA-TAT>BR>VC>Pro>GP > pTyr294aa.

## Discussion

WASP regulates sealing ring formation through regulatory mechanisms mediated by Rho family GTPases (Rho and Cdc42), c-Src kinase, and a phosphatase PTP-PEST [20]. WASP null osteoclasts failed to demonstrate sealing ring, and are defective in bone resorption [8]. To identify the domain of WASP that is critical for sealing ring formation, we transduced TAT-fused peptides containing different domains of WASP into osteoclasts. 'V' and 'C' domains of WASP are essential to induce actin polymerization through their binding to actin monomers and Arp2/3 complex, respectively [29]. Interaction of Arp2/3 with the activated WASP nucleates and cross-links actin filaments [30,31]. In osteoclasts transduced with 'VC' domain, sealing ring formation is reduced (Fig. 6G). The sealing rings are smaller than the ones observed in osteoclasts transduced with control proteins (HA-TAT or HSV-TK; Fig. 6B, and 6H). These osteoclasts also exhibited actin clumps or aggregates in the center. Our results are in agreement with those of Hufner et al., who showed that microinjection of both GST-VC and GST-CA induced actin accumulations in primary human macrophages or human endothelial vein cells. These actin accumulations also displayed colocalization of Arp2/3 [19]. Together, these results indicate that transduced or microinjected peptide competitively inhibited Arp2/3 binding to endogenous WASP.

Our data show that the inhibitory effect with the TAT-fused WASP peptide was observed in the following order: pTyr294 = GP domain>Pro>BR = VC = VCA. The inhibitory effect of TAT-fused peptides containing BR (Fig. 6D and 7D), and VC (Fig. 6G and 7H) domains on sealing ring formation and bone resorption may be transitory. The observed actin polymerization (Fig. 3) and sealing ring formation (Fig. 6D and 6G) in these osteoclasts may have been caused by either the pre-existing Arp2/3-WASP networks or resumed activation of endogenous WASP after the partial inhibitory effects were mediated by these peptides.

The consequence of transduction of pro-rich WASP peptide was apparent in the abnormality of sealing ring structures (Fig. 6E, and 6E'). Our studies show that transduction of pro-rich WASP peptide resulted in the formation of nascent podosome ring or sealing ring-like structures throughout the osteoclasts. This is comparable to the effect observed in osteoclasts treated with phenyl arsine oxide (PAO), a phosphatase inhibitor. Previous studies on PTP-PEST function have shown that its activity is essential for the interaction of signaling proteins with WASP or gelsolin. This was confirmed in osteoclasts treated with PAO or SiRNA to PTP-PEST [20,25]. Similar inhibitory effect appears to take place in cells transduced with the WASP peptide containing pro-rich region. The avidity of this peptide to signaling molecule is more as it contains six proline-rich clusters (307–428aa): 311–318 (8), 350–355(6), 358–361 (4), 366–372 (7), 379–385 (7), and 390–403 (14). Although this peptide did not reduce the extent of the spreading of osteoclasts, it affected typical sealing ring formation in resorbing osteoclasts (Fig. 6E, and 6E'). These results provide a link between the proline-rich sequences and the regulation of sealing ring formation in osteoclasts. It is possible that inhibition of signal complex formation with WASP or gelsolin may reduce the spatially and temporally regulated functions of these proteins in the organization of podosomes or sealing ring [20]. However, the significant inhibition of WASP activation by the peptide containing pTyr294 suggests that phosphorylation and subsequent interaction of signaling proteins with WASP proline rich sequences may cause actin polymerization for extended time by Arp2/3 complex. The actin polymerization by Arp2/3 complex is augmented by tyrosine or serine phosphorylation of WASP [10,11,15,32,33].

Next, the questions are, How does phosphorylation of WASP influence WASP mediated events? How does the WASP peptide containing pTyr294aa block the osteoclast function? It has been suggested that phosphorylation of human WASP at Y291 can effect two important aspects. First, the WASP, which is phosphorylated on pTyr291, has higher basal activity. Second, it could enable protein-protein interactions by SH2-SH3 binding and respond to new signal through complex interaction of signaling proteins [34]. We have previously shown that addition of osteopontin to osteoclasts increases interaction of SH2-SH3 containing signaling molecules with phosphorylated WASP (p-WASP) and sealing ring formation through integrin αvβ3-mediated signaling mechanism [20]. This complex formation is reduced in osteoclasts transduced with dominant negative Src [20].

Phosphorylation of N-WASP on Tyr256 corresponds to the Tyr291 in WASP. Phosphorylation of N-WASP and its consequent localization in the cytoplasm increased the ability of N-WASP to stimulate cell migration [10]. FAK slightly increased N-WASP as compared to Fyn, which exhibited a strong stimulation [35]. The differences in the effects mediated by FAK and Src family kinases may be due to the presence of SH2 and SH3 domains in Src kinases [34]. Binding of Src family kinases to WASP family proteins has several functional consequences in different cell systems. Inhibition of phosphorylation with a selective Src family kinase inhibitor prevents N-WASP mediated neurite outgrowth [35]. Wu et al. suggested that binding of SH2 and SH3 containing proteins is more responsible for the stimulation of N-WASP activity than phosphorylation of N-WASP [10].

However, in the present study, we could show that phosphorylation of pTyr294aa is essentially responsible for WASP-mediated effects in osteoclasts. This is consistent with the observation that WASP phosphorylation is critical for T cell activation. Over expression of pTyr mutant (Y291F) or inhibition of Src family kinases affects T-cell activation or neurite outgrowth, respectively [12,35]. WASP peptide containing pTyr294 not only competitively blocked the phosphorylation of endogenous WASP but also its interaction with c-Src (Fig. 5) and other signaling proteins (data not shown). Inhibition of c-Src interaction with endogenous WASP by both proline-rich and pTyr294aa containing WASP peptide raised the possibility that phosphorylation of WASP could be the first step in the formation of multimeric signaling complex with WASP [20]. Phosphorylation of WASP and the subsequent interaction of signaling proteins with WASP through proline-rich clusters (311–404) are critical for WASP-mediated effects in osteoclasts.

The process of sealing ring formation requires phosphorylation events arbitrated by tyrosine kinases [20,36,37]. From our earlier [2,20] and present observations, it is tempting to speculate that phosphorylation of pTyr294 contributes to complex interaction of signaling molecules with WASP. This will result in cytoskeletal events leading to the formation of sealing ring during bone resorption. Multiple mechanisms may control downstream events, including phosphorylation and interaction of signaling proteins with WASP to enhance osteoclast function. As suggested by Torres and Rosen [34], it is plausible that the phosphorylation and dephosphorylation events may provide mechanisms to achieve continual actin remodeling process during bone resorption. Sealing ring formation may be achieved through the formation of signaling complex by SH2-SH3 interactions. The formation of signaling complex may not only increase the half-life of the phosphorylated WASP but also prevents it from dephosphorylation by phosphatase(s). Furthermore, WASP phosphorylation by Src family kinases plays a role in stabilizing the unmasked conformation of WASP following the decay of GTPase signals [34] and increases its affinity for Arp2/3 complex [15,35]. Competitive inhibition of phosphorylation of endogenous WASP by the transduced pTyr294 peptide (Fig. 5E) may ultimately block the above-mentioned events which are critical for sealing ring formation.

However, it will be of interest to further elucidate how phosphorylation of pTyr294aa modulates WASP function in osteoclasts. The focus of future studies would be on the mechanisms by which WASP-peptide containing pTyr294 regulates the activity of WASP in sealing ring formation. Once the mechanisms are understood, therapeutic measures can be developed with this pTyr294aa containing peptide to reduce bone resorption during osteoporosis caused by estrogen deficiency or cancer metastasis in general.

## Conclusion

Based on our observations, we suggest that modulation of phosphorylation state of pTyr294aa in WASP or binding of SH3 containing signaling proteins with the proline-rich region of WASP may assist in integrating multiple signaling pathways that partake in the assembly of sealing ring and bone resorption. These biologically active permeable WASP peptides containing pro-rich region and pTyr294aa function as inhibitors of sealing ring formation and bone resorption activity of osteoclasts. Thus, these studies suggest that WASP and WASP-based signaling complex of the osteoclast sealing ring is an attractive target for pharmacologic regulation of bone resorption.

## Abbreviations

WASP, Wiskott-Aldrich Syndrome protein; HA, hemagglutinin; TAT, transactivator peptide with transforming properties; RANKL, receptor activator of nuclear factor-kappa B ligand; mCSF-1, macrophage colony stimulating factor; HSV-TK, herpes simplex virus-thymidine kinase; PTP-PEST, protein tyrosine phosphatase-proline-glutamic acid, serine, threonine amino acid sequences; VCA, verpolin, cofilin, acidic domain; aa-amino acid; GP, GTPase binding (G) and proline-rich (P) domain; F-actin, filamentous actin; FL, full length; Arp2/3; actin-related proteins 2 and 3; NI, non-immune serum

## Competing interests

The author(s) declare that they have no competing interests.

## Authors' contributions

TM participated in the design of the study as well as carried out the biochemical and cell biological studies. VS cloned and purified pTyr291 and pro-rich WASP domains. MAC conceived of the study, participated in its design, and drafted the manuscript. All authors read and approved the final version of the manuscript.
